# Supplementary material for: Comprehensive Evaluation of Hepatotoxicity Following Radiation Therapy in Breast Cancer Patients
Source: Cancers (Basel). 2025 Oct 8;17(19):3252. doi: 10.3390/cancers17193252 (PMC12523911; doi:10.3390/cancers17193252)
Supplement: Supplementary file 1 [file cancers-17-03252-s001.zip › cancers-3885983-supplementary.pdf]

Supplementary Materials

Figure S1. A representative case of breast cancer patient showing clinical target volume delineated according to either the RTOG or ESTRO contouring guideline.

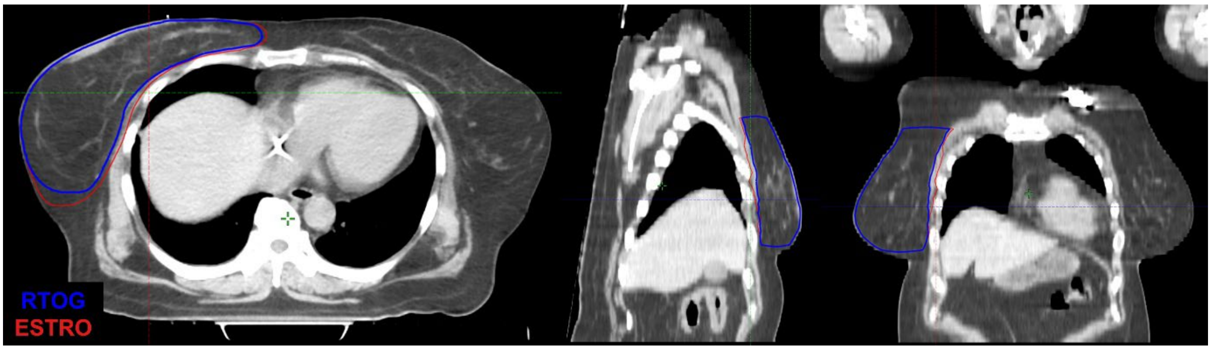

Table S1 Estimation of the optimal cutoff of DVH parameters

	Optimal cutoff	Sn	Sp	AUC
**V5Gy**	3.54	0.74	0.44	0.59
**V10Gy**	1.74	0.54	0.58	0.56
**V20Gy**	0.01	0.69	0.44	0.56
**Mean Dose**	135.2	0.74	0.44	0.59

Abbreviations: AUC, Area under the curve; DVH, Dose-volume histogram; Sp, Specificity; Sn, Sensitivity; VnGy, Volume of liver receiving n Gy or greater.

Table S2 Comparative DVH analysis of four representative cases planned using both RTOG and ESTRO guidelines

Patient	RTOG				ESTRO				Change (%)			
	**Mean Dose (cGy)**	**V5Gy (%)**	**V10Gy (%)**	**V20Gy (%)**	**Mean Dose (cGy)**	**V5Gy (%)**	**V10Gy (%)**	**V20Gy (%)**	**Mean Dose (cGy)**	**V5Gy (%)**	**V10Gy (%)**	**V20Gy (%)**
**A**	859.7	77.2	29.9	4.9	862.6	74.5	31.4	5.2	**0.3%**	**-3.4%**	**5.2%**	**6.8%**
**B**	849.2	75.6	30.9	3.1	847.4	73.7	32.0	3.1	**-0.2%**	**-2.5%**	**3.8%**	**0.8%**
**C**	860.8	71.3	32.7	3.0	818.1	66.2	30.8	3.1	**-5.0%**	**-7.1%**	**-5.6%**	**4.4%**
**D**	875.4	70.0	24.8	8.0	866.9	70.1	23.3	7.9	**-1.0%**	**0.2%**	**-5.9%**	**-1.4%**
**AVG**	861.3	73.5	29.6	4.7	848.8	71.1	29.4	4.8	**-1.5%**	**-3.2%**	**-0.5%**	**2.0%**

Abbreviations: ESTRO, European Society for Radiotherapy and Oncology; RTOG, Radiation Therapy Oncology Group; VnGy, Volume of liver receiving n Gy or greater.
